# Successful transseptal puncture through tortuous inferior vena cava with a novel transseptal guidewire: a case report

**DOI:** 10.1093/ehjcr/ytaf512

**Published:** 2025-10-07

**Authors:** Tongshuai Chen, Yuhang Yang, Bing Rong, Kai Zhang, Jingquan Zhong

**Affiliations:** State Key Laboratory for Innovation and Transformation of Luobing Theory, Key Laboratory of Cardiovascular Remodeling and Function Research of MOE, NHC, CAMS and Shandong Province, Department of Cardiology, Qilu Hospital of Shandong University, 107 Wenhua Xilu, Jinan, Shandong 250012, China; State Key Laboratory for Innovation and Transformation of Luobing Theory, Key Laboratory of Cardiovascular Remodeling and Function Research of MOE, NHC, CAMS and Shandong Province, Department of Cardiology, Qilu Hospital of Shandong University, 107 Wenhua Xilu, Jinan, Shandong 250012, China; State Key Laboratory for Innovation and Transformation of Luobing Theory, Key Laboratory of Cardiovascular Remodeling and Function Research of MOE, NHC, CAMS and Shandong Province, Department of Cardiology, Qilu Hospital of Shandong University, 107 Wenhua Xilu, Jinan, Shandong 250012, China; State Key Laboratory for Innovation and Transformation of Luobing Theory, Key Laboratory of Cardiovascular Remodeling and Function Research of MOE, NHC, CAMS and Shandong Province, Department of Cardiology, Qilu Hospital of Shandong University, 107 Wenhua Xilu, Jinan, Shandong 250012, China; State Key Laboratory for Innovation and Transformation of Luobing Theory, Key Laboratory of Cardiovascular Remodeling and Function Research of MOE, NHC, CAMS and Shandong Province, Department of Cardiology, Qilu Hospital of Shandong University, 107 Wenhua Xilu, Jinan, Shandong 250012, China; Department of Cardiology, Qilu Hospital (Qingdao), Cheeloo College of Medicine, Shandong University, Qingdao, Shandong 250012, China

**Keywords:** Atrial fibrillation, Transseptal puncture, Inferior vena cava tortuosity, Case report

## Abstract

**Background:**

Atrial transseptal puncture is a fundamental manoeuvre in transseptal transcatheter interventions, and inferior vena cava tortuosity could cause failure of conventional transseptal puncture method with Brockenbrough needle.

**Case summary:**

We report a rare case of inferior vena cava tortuosity in a patient with atrial fibrillation in which the conventional transseptal puncture method failed due to marked needle deformation and was completed by a novel transseptal wire (AccuSafe™, Synaptic Medical) in combination with a steerable sheath.

**Discussion:**

Inferior vena cava tortuosity is one of the major causes of unsuccessful atrial transseptal puncture, even under the guidance of intracardiac echocardiography. The new transseptal wire should be the initial selection rather than a conventional transseptal needle when encountering severe inferior vena cava tortuosity.

Learning points

- In the case of inferior vena cava tortuosity, transseptal puncture is very difficult with conventional Brockenbrough needle.
- The new transseptal wire should be the initial selection rather than a conventional transseptal needle when encountering severe inferior vena cava tortuosity.

## Introduction

Tortuosity of the inferior vena cava (IVC) may be incidentally discovered during ablation procedures in atrial fibrillation (AF) patient, introducing intraoperative challenges. We present the case of a patient with failed transseptal puncture (TSP) via Brockenbrough (BRK) needle caused by IVC tortuosity, and successful TSP achieved by a new type atrial septal puncture guidewire finally.

## Summary figure

**Figure:**
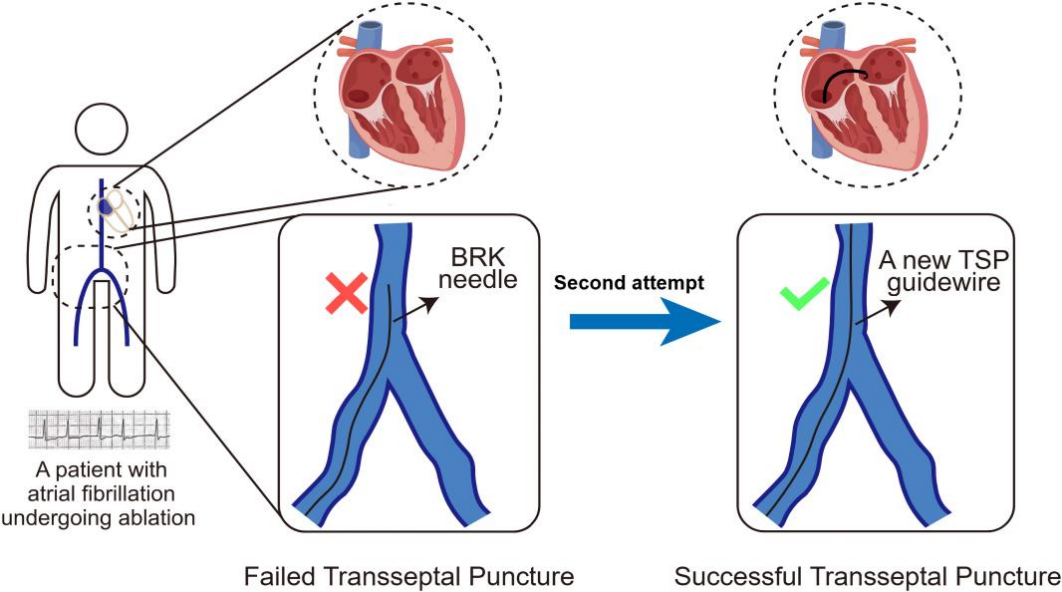


## Case presentation

**Figure 1:**
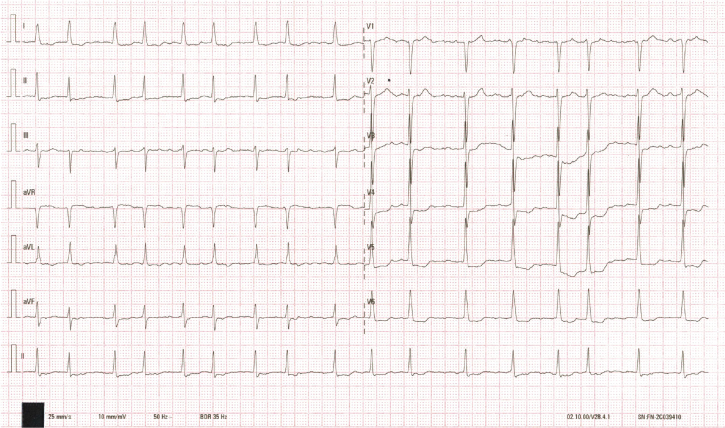
The 12-lead electrocardiogram upon the patient’s admission showed atrial fibrillation with rapid ventricular response.

**Figure 2:**
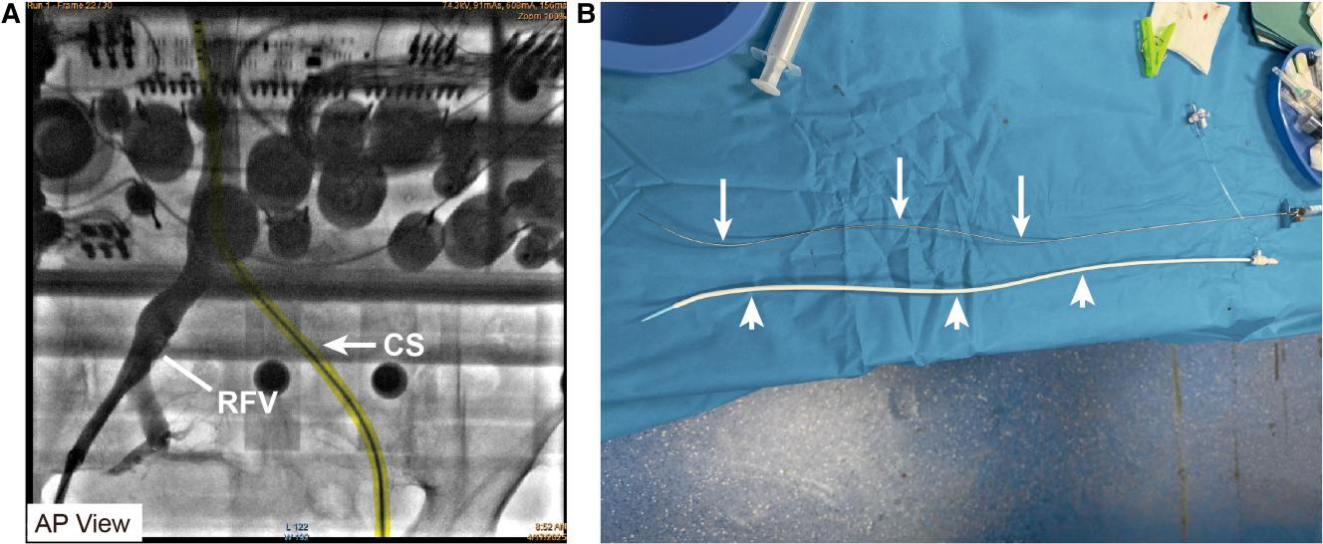
Failed first attempt at transseptal puncture. (*A*) Unsuccessful attempt of transseptal puncture due to tortuosity of the right femoral vein (short white line), and CS was advanced in the left femoral vein (yellow marking and white arrow). (*B*) Multiple curvatures on different planes of Brockenbrough needle (upper, long white arrows) and SL1 sheath (lower, short white arrows) showed the inferior vena cava tortuosity. RFV, right femoral vein; CS, coronary sinus catheter.

**Figure 3:**
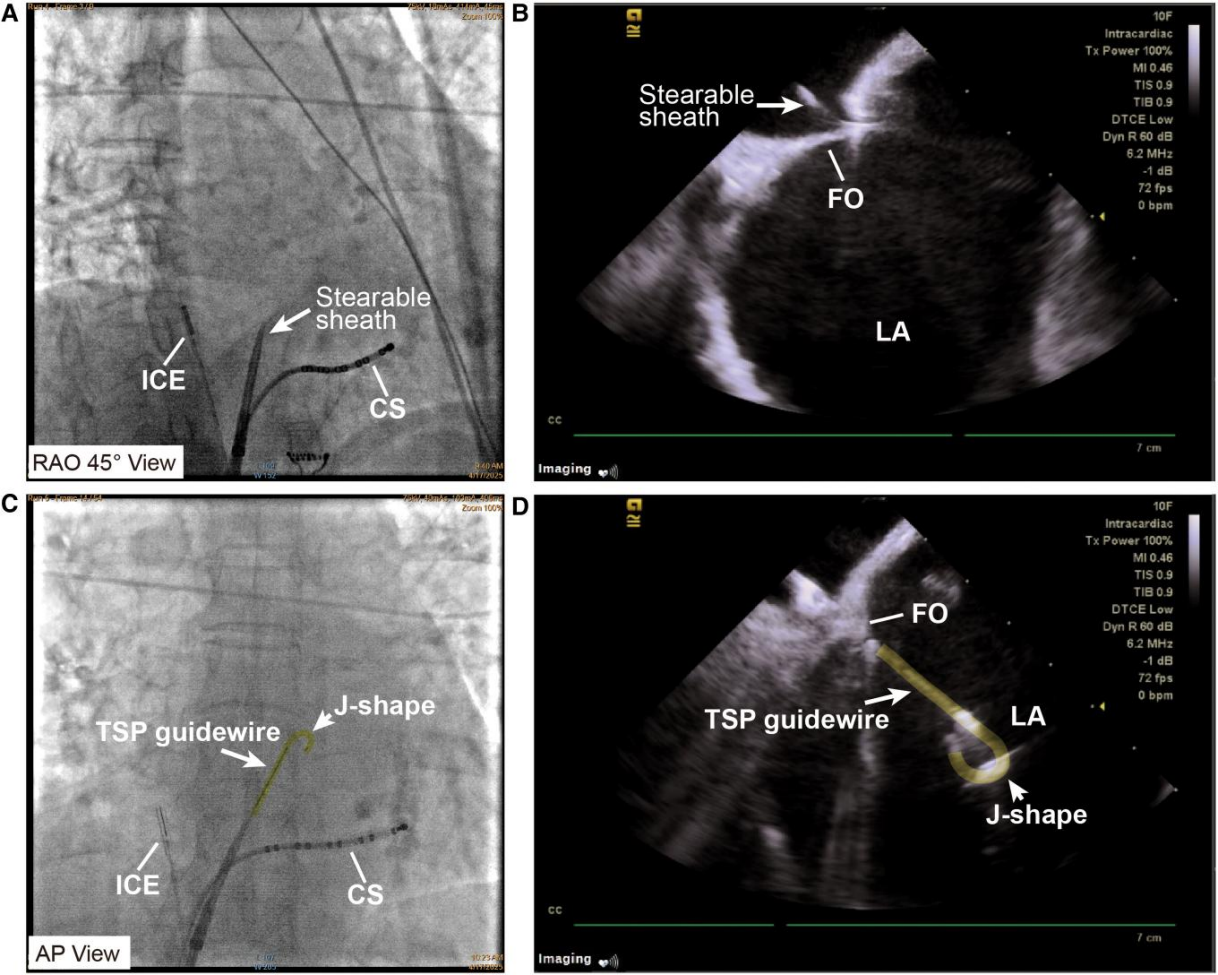
Successful transseptal puncture procedure with a novel transseptal wire. (*A* and *B*) A steerable sheath (CARTO VIZIGO™, Biosense Webster, USA) was advanced to the fossa ovalis under X-ray fluoroscopy (*A*, white arrow) and intracardiac echocardiography guidance (*B*, white arrow). (*C* and *D*) Attempts of transseptal puncture with a transseptal puncture guidewire (AccuSafe™, Synaptic Medical, yellow markings). A ‘J shape’ (short white arrows) formed in the distal part of wire (long white arrows) automatically after transseptal puncture, which was observed clearly under X-ray fluoroscopy (*C*) and intracardiac echocardiography (*D*). CS, coronary sinus catheter; FO, fossa ovalis; ICE, intracardiac echocardiography; LA, left atrium; TSP, transseptal puncture.

**Figure 4:**
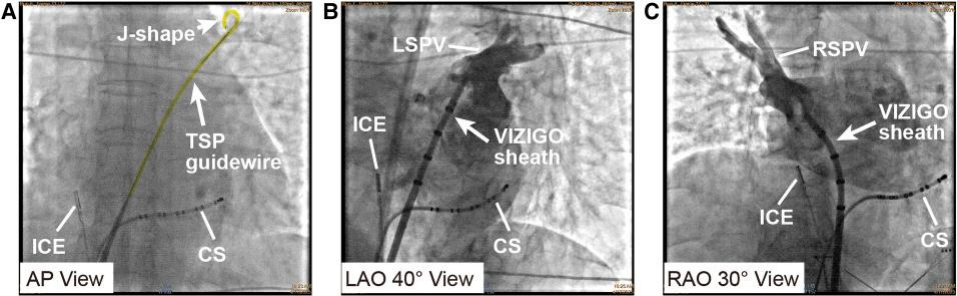
Introducing the sheath into the left atrium. (*A*) The transseptal puncture guidewire (yellow marking and long white arrow) was introduced into the left atrium and reached left superior pulmonary vein (LSPV) safely with a ‘J shape’ (short white arrow). (*B*) Left superior pulmonary vein angiography was performed with VIZIGO sheath (long white arrow) introduced into the left atrium by transseptal puncture wire. (*C*) Right superior pulmonary vein angiography was performed with VIZIGO sheath (long white arrow). CS, coronary sinus catheter; ICE, intracardiac echocardiography; LSPV, left superior pulmonary vein; RSPV, right superior pulmonary vein; TSP, transseptal puncture.

A 73-year-old woman was consented for persistent AF radiofrequency ablation (*Figure 1*). The patient’s informed consent was obtained. Local anaesthesia and deep sedation were applied during procedure. The first attempt at TSP was from the right femoral access, it was unsuccessful because of severe femoral vein and iliac vein tortuosity, so the SL1 sheath SL1 sheath (Abbott, USA) cannot go through right femoral vein and iliac vein and failed to reach the IVC (*Figure 2A*). The second attempt was from the left femoral access, SL1 sheath was advanced on a supporting guidewire to the superior vena cava (SVC), and then the BRK needle was inserted inside the dilator stiffly, but cannot reach the tip level of SL1 sheath. High resistance to BRK needle (Abbott, USA) advancement into dilator was felt, and the patient complained of low back pain when the BRK needle was inserted; when removed, the BRK needle showed multiple curvatures on different planes as consequence of the IVC tortuosity (*Figure 2B*). Attempts of TSP with an 8.5-Fr steerable sheath (CARTO VIZIGO™, Biosense Webster, USA) and a new type atrial septal puncture guidewire (AccuSafe™, 0.032in*230 cm, Synaptic Medical) were tried to overcome tortuosity of femoral and IVC (*Video 1*). Unlike SL1 sheath, the steerable sheath was easier to reach IVC via right femoral vein and then withdrawn while rotating into fossa ovalis (FO) under guide of intracardiac echocardiograph (ICE) image (*Figures 3A* and *B* and *Video 2*). A TSP was performed by delivering a slight mechanical push of transseptal wire. Then, the transseptal wire was introduced into left superior pulmonary vein (LSPV) safely because the distal part of wire formed ‘J shape’ automatically (*Figures 3C* and *D* and *Video 3*). Subsequently, the Vizigo sheath was introduced into the left atrium (LA), and the AF ablation procedure was completed without complications (*Figure 4*). The patient has not reported any related complications or AF recurrence during 2 months approximately after the ablation.

## Discussion

Transseptal puncture performed with the BRK needle is technically demanding and carries potential risks such as cardiac perforation. Access from femoral vein and IVC is feasible for most case with conational use of a rigid, metal BRK needle and SL1 sheath^1^; however, the long and rigid needle may tear the surrounding vein tissue and cause life-threatening complications due to its bulkiness and difficulty in operation. Previous studies have emphasized the risk of needle puncture in tortuous veins.^2^

Congenital variants of the IVC emerge as a result of different alterations in this developing process. These anomalies are uncommon, usually of little physiological consequence, and mostly discovered incidentally in healthy individuals during cross-sectional imaging.^3^ However, they do have implications of relevance to operations, because they may lead to significant complications during vascular interventional radiology procedures.^4^

Because few 3D reconstruction of preoperative cross-sectional imaging of IVC is applied according to present AF ablation practice guidelines,^5^ peri-operative finding of IVC high tortuosity could bring risk to TSP failure. Previous animal study introduced an alternative transseptal method using the backend of percutaneous transluminal coronary angioplasty (PTCA) guidewire and microcatheter; however, the stiff backend of PTCA wire could be high risky for perforation of the LA.^6^

**Figure 5:**
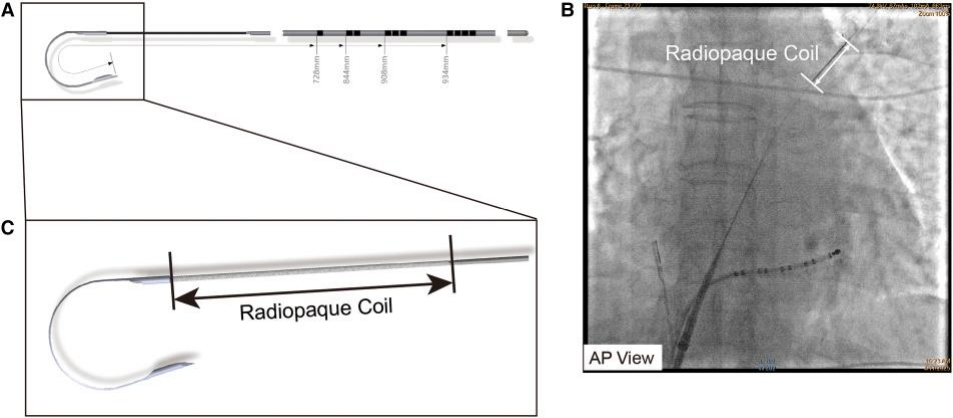
AccuSafe™ guidewire (Synaptic Medical). (*A*) After passing through the septum, the wire can form a ‘J shape’ automatically. (*B*) The radiopaque coil in the distal tip of the guidewire can be visualized under X-ray fluoroscopy (white arrow). (*C*) Enlarged image of the radiopaque coil made of platinum–tungsten alloy (black arrow).

In this case, a new type of transseptal wire (AccuSafe™, Synaptic Medical) was adopted to overcome IVC tortuosity. This transseptal wire has been reported to be more feasible to overcome TSP difficulty when encountering giant atrial septum aneurysm.^7^ Unlike rigid BRK needle, the AccuSafe wire can go through tortuous part of IVC smoothly. The transseptal wire has a nitinol wire with a diameter of less than 0.020 in, featuring a sharp distal tip and a preforms ‘J’ shape, with the dimensions shown in *Figure 5A*. The distal wire tip has a radiopaque coil to enable fluoroscopic localization (*Figure 5B*), seen in magnified view in *Figure 5C*. When the wire breaks through FO, the sharp distal can form ‘J’ shape immediately, allowing it to be safely advanced into the LSPV without the need to exchange the wire. Besides, other type of energy could be applied with the transseptal wire methods. Jaffar *et al.* used a technique by electrifying a coronary guidewire when transseptal needle puncture is unsuccessful. A coronary guidewire, connected to an electrosurgery pencil, was advanced through the *trans*-septal needle, dilator, and sheath to perforate the interatrial septum during a short burst of radiofrequency energy.^8^ When encountering IVC with high tortuosity, a special guidewire with brief radiofrequency energy delivering could also be safe and effective. Imaging tool like ICE was necessary for TSP procedure operated with transseptal wire. Firstly, when the TSP system (including steerable TS sheath with transseptal wire) was withdrawn from the SVC to the midpoint fossa ovals, the above process can be monitored by ICE clearly. Secondly, when the transseptal wire was extended outside the steerable sheath and advanced across the interatrial septum, ICE can monitor the process clearly which is believed to improve the procedure safety. Another prospective study tried to testify feasibility of TSP-guidewire in patients with a resistant atrial septum. In this study, the criterion to use the TSP-guidewire was a resistant atrial septal, defined as inability to perforate the FO by applying moderate pressure to a standard BRK needle. Nineteen patients (23%) in 27 procedures showed a resistant atrial septum. In all these procedures, the TSP-guidewire was safely and successfully used to accomplish the TSP procedure.^9^ Therefore, we believe that the new transseptal wire should be initial selection rather than a conventional septal puncture tool when encountering anatomical abnormalities such as vein with high tortuosity or abnormal atrial septum structure.

## Lead author biography


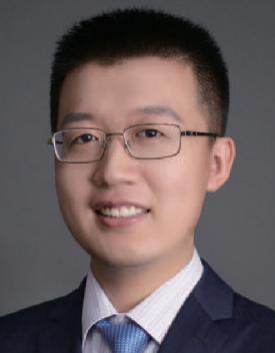


Dr Tongshuai Chen is a cardiac electrophysiologist with 10+ years of experience in tachyarrhythmia management, during his cardiology fellowship; he did research on the genetics of hypertrophic cardiomyopathy in Key Laboratory of Cardiovascular Remodeling and Function Research of MOE, NHC, CAMS, and Shandong Province; and his clinical interests include diagnosis and management of tachyarrhythmia disorders and ablation for supraventricular and ventricular tachycardias.

## Data Availability

Further information is available from the corresponding author.
